# Hispanic-Latino Race is Associated with Worse Heart Failure Symptoms in Patients with Hypertrophic Cardiomyopathy

**DOI:** 10.31083/RCM26588

**Published:** 2025-02-20

**Authors:** Reshma Golamari, Alexander Khodak, Steven D. Hartman, Marissa Donatelle, Tarec K. Elajami, Rafle Fernandez, Christos G. Mihos

**Affiliations:** ^1^Columbia University Irving Medical Center, Division of Cardiology, Mount Sinai Heart Institute, Miami Beach, FL 33140, USA; ^2^Department of Internal Medicine, Mount Sinai Medical Center, Miami, FL 33140, USA; ^3^Echocardiography Laboratory, Division of Cardiology, Mount Sinai Heart Institute, Columbia University Irving Medical Center, Miami Beach, FL 33140, USA

**Keywords:** epidemiology, ethnicity, heart failure, hypertrophic cardiomyopathy

## Abstract

**Background:**

Data regarding racial differences in patients with hypertrophic cardiomyopathy (HCM) is sparse. We hypothesized that Hispanic-Latino (HL), Non-Hispanic (NH), and African-American (AA) race impacts the clinical presentation of HCM.

**Methods:**

A total of 641 HCM patients (HL = 294, NH = 274, AA = 73) were identified retrospectively from our institutional registry between 2005–2021. Clinical characteristics, echocardiographic indices, and outcomes were assessed using analysis of variance, Kruskal-Wallis, and multivariate linear regression statistical analyses, with Dunn-Bonferroni and Tukey test applied in post-hoc pairwise assessments.

**Results:**

The HL and NH patients were older compared with AA (69.2 ± 14.7 vs 67.9 ± 15.3 vs 59.4 ± 15.8 years; *p* < 0.001). The HL group had higher prevalence of females compared with NH (62 vs 47%; *p* = 0.002), and more moderate-severe mitral regurgitation (35 vs 23 vs 12% *p* < 0.001) and a higher E/e’ ratio (16.4 ± 8.1 vs 14.9 ± 6.6 vs 13.3 ± 4.5; *p* = 0.002) when compared with NH and AA. Multivariate linear regression analysis revealed HL ethnicity (β = 0.1) was associated with worse New York Heart Association (NYHA) class independent from moderate-severe mitral regurgitation (β = 0.2), chronic obstructive pulmonary disease (β = 0.17), female gender (β = 0.13), coronary artery disease (β = 0.12), atrial fibrillation (β = 0.11), peak trans-mitral E-wave velocity (β = 0.11), left ventricular mass index (β = 0.1), and reverse septal curve morphology (β = 0.1) (model, r = 0.5, *p* < 0.001). At 2.5-year median follow-up, all-cause mortality (8%) and composite complications (33%) were similar across the cohort.

**Conclusions:**

HCM patients of HL race have worse heart failure symptoms when compared with NH and AA, with severity independent of cardiovascular co-morbidities.

## 1. Introduction 

Hypertrophic cardiomyopathy (HCM) is a heterogeneous clinical disorder with a variable expression, and an estimated prevalence of 1:200 to 1:500 individuals [1, 2]. Racial and ethnic differences along the disease spectrum are recognized as these disparities may impact the clinical presentation and outcomes [3]. The prevalence of HCM amongst racial and ethnic groups varies, with 8–13% noted in African-Americans (AA) versus 87–92% in Non-Hispanic (NH) White patients [4, 5]. In addition to being underrepresented in clinical investigations, Hispanic-Latino (HL) and AA communities have been demonstrated to have worse cardiovascular risk profiles and experience inadequacies in care. These inadequacies include lower rates of implantable cardioverter defibrillator placement and septal reduction procedures when compared with NH White patients [3, 6].

Important phenotypic differences by echocardiography and cardiac magnetic resonance imaging have been suggested between NH Whites and AA [7]. This includes a greater prevalence of neutral and apical left ventricular (LV) dominant hypertrophy and less obstructive physiology, with similar LV ejection fraction, chamber size, and myocardial fibrosis in AA patients [3, 7]. The contrasting clinical presentation of the groups remains less understood, and salient comparisons with HL populations are lacking. We hypothesized that HL, NH, and AA race may differentially impact the clinical presentation and course of HCM, and sought to provide detailed echocardiographic and outcomes assessments and comparisons across these patient populations.

## 2. Materials and Methods

### 2.1 Patient Selection

The Mount Sinai Medical Center/Mount Sinai Heart Institute (Miami Beach, FL, USA) Institutional Review Board approved the study protocol, which was drafted and structured in accordance with the 1975 declaration of Helsinki guidelines (revised in 2013). Adult patients ≥18 years old with HCM followed at our institution were retrospectively collated from our digital echocardiography HCM database, which includes inpatient and outpatient studies, between January 2005 and December 2021. All echocardiograms were reviewed by two level III board-certified echocardiographers for confirmation of the diagnosis (RF, CGM). Patients were stratified according to their self-reported race as HL, NH, and AA. The NH group consisted of patients who identified as White, Asian, or Other. Variables were collected via careful review of each individual’s electronic health record.

A diagnosis of HCM required: (1) a LV wall thickness ≥15 mm at end-diastole; (2) a septal-to-posterior wall (or apical-to-posterior wall in apical HCM) thickness ratio >1.5; and, (3) the absence of another pathologic or physiologic cause of hypertrophy [8]. All patients meeting the HCM criteria were screened for phenocopy conditions, and careful assessment of anti-hypertensive control was required. More specifically, patients meeting clinical and/or multi-parametric imaging diagnostic criteria for any of the following pathologies were excluded: (1) untreated or uncontrolled hypertension; (2) hypertensive heart disease; (3) infiltrative cardiomyopathy (i.e., cardiac amyloidosis, sarcoidosis); (4) phenocopy conditions (i.e., Andersen-Fabry disease, Danon disease, Friedrich’s ataxia). When performed or available, ancillary imaging studies such as cardiac magnetic resonance imaging and nuclear single-photon emission computerized tomography were reviewed.

Obstructive HCM was defined as a peak systolic LV outflow tract pressure gradient of ≥30 mmHg either at rest or with provocation. HCM phenotype was classified according to the Mayo Clinic criteria as follows: (1) sigmoid septum—pronounced basal septal bulge concave to the LV cavity; (2) reverse curve—crescent-shaped cavity with predominantly mid-to-distal concave septal hypertrophy; (3) apical—hypertrophy predominantly distal to the papillary muscle insertions with an ‘ace of spades’ morphology; and, (4) neutral—septal hypertrophy extending to the posterior wall with minimal LV convexity or concavity [9].

### 2.2 Echocardiographic Analysis

A GE Vivid E9, E95 or S70 cardiovascular ultrasound system (General Electric Healthcare, Waukesha, WI, USA) was utilized to perform all transthoracic echocardiographic examinations. The assessment of cardiac geometry and function was performed in accordance with the American Society of Echocardiography (ASE) chamber quantification guidelines [10]. Specifically, maximal interventricular septal and posterior LV wall thickness was measured at end-diastole in the parasternal long-axis view at the level of the mitral valve leaflet tips. The maximal apical LV wall thickness was assessed in the three standard apical views and in a cross-sectional parasternal short-axis view distal to the papillary muscle insertions. The ASE recommendations for the evaluation of LV diastolic function were applied to estimate LV compliance, relaxation, and filling pressure [11]. Systolic anterior motion (SAM) of the mitral valve and mitral valve regurgitation severity were assessed in accordance with the ASE recommendations for noninvasive evaluation of native valvular regurgitation. A multi-parametric method was utilized to grade the severity of mitral regurgitation as none/trace, mild, moderate, and severe [12].

### 2.3 Study Endpoints

Heart failure (HF) symptomatology was assessed using the New York Heart Association (NYHA) functional class. The primary endpoint of the study was to assess the impact of ethnicity, demographic, clinical, and echocardiographic variables on NYHA class in patients with HCM. The secondary endpoint of the study was the composite outcome of all-cause mortality or any cardiovascular hospitalization at follow-up. Individual clinical endpoints included cardiovascular mortality, sudden cardiac death, myocardial infarction, cerebrovascular accident, incidence of septal myectomy or alcohol septal ablation, or hospitalization for HF, angina, or arrhythmia.

### 2.4 Statistical Analysis

Data was analyzed using IBM SPSS Statistics version 21 (IBM Corporation, Armonk, NY, USA). Categorical variables were expressed as number (frequency), while continuous variables were expressed as mean (± standard deviation) or median (interquartile range, IQR) dependent upon their Gaussian distribution. Categorical variables were compared using a Kruskal-Wallis test, with the Dunn-Bonferroni post-hoc test performed to assess for specific inter-group differences. Continuous variables were analyzed using the one-way analysis of variance test, with the Tukey post-hoc test performed to assess for specific inter-group differences. Isolated differences between two groups found in the exploratory post-hoc analyses were specifically reported within the context of the primary hypothesis. Hierarchical multivariable linear regression analysis tested for correlations between demographic, clinical, and echocardiographic measures, with NYHA functional class; step one included the constant and HL race, and step two incorporated exploratory variables whose univariate correlations with NYHA functional class were statistically significant. Ordinal regression is performed only when meeting the assumptions of no multi-collinearity, and having the existence of proportional odds. The range estimates and expected treatment effects are expressed as unstandardized and standardized beta coefficients, with a 95% confidence interval. A *p*-value < 0.05 was considered statistically significant.

## 3. Results

### 3.1 Patient Demographics and Clinical Characteristics

A total of 641 patients were identified, of which 294 (46%) were HL, 274 (43%) were NH, and 73 (11%) were AA, respectively. Females comprised 54% of the cohort, and the most common co-morbidities were hypertension (83%), diabetes mellitus (26%), and coronary artery disease (23%). An implantable cardioverter defibrillator was present in 54 (8%) patients. Six patients (1%) had a history of septal myectomy, and 8 (1%) had a prior percutaneous alcohol septal ablation. The median follow-up was 2.5 (IQR, 0.5–6.1) years and was 100% complete.

Comparisons are presented between the HL, NH, and AA groups, with post-hoc analyses included as warranted. Patients in the HL group had a higher NYHA functional class when compared with NH and AA patients (1.8 ± 0.8 vs 1.6 ± 0.7 vs 1.3 ± 0.5, *p* < 0.001). Additionally, HL patients had a greater prevalence of female gender (62 vs 47%, *p* = 0.002) and used aspirin more often than NH individuals (48 vs 36%, *p* = 0.008), and experienced more NYHA class III or IV symptoms when compared with AA (19 vs 4%, *p* = 0.004). AA patients were younger (69.2 ± 14.7 vs 67.9 ± 15.3 vs 59.4 ± 15.8 years, *p* < 0.001), and when compared with HL patients had a higher mean diastolic blood pressure (73 ± 12 vs 77 ± 12 mmHg, *p* = 0.03). A greater prevalence of diabetes mellitus was noted in the NH group when compared with HL and AA (31 vs 18 vs 38%, *p* < 0.001), and NH patients were also noted to have a greater prevalence of chronic obstructive pulmonary disease when compared with AA (12 vs 1%, *p* = 0.01). No other differences in patient demographics and clinical characteristics were observed (Table 1).

**Table 1.** **Demographics and clinical characteristics of patients with hypertrophic cardiomyopathy according to ethnicity**.

Variable		Hispanic	Non-Hispanic	African-American	*p*-value
		N = 294	N = 274	N = 73	
Age ^a,b^		69.2 ± 14.7	67.9 ± 15.3	59.4 ± 15.8	<0.001
Female ^c^		182 (62%)	130 (47%)	35 (48%)	0.001
Heart rate (beats/minute)		75 ± 15	73 ± 16	77 ± 16	0.14
Systolic blood pressure (mmHg)		130 ± 19	131 ± 19	135 ± 20	0.25
Diastolic blood pressure (mmHg) ^d^		73 ± 12	74 ± 12	77 ± 12	0.03
Glomerular filtration rate		73 ± 31	75 ± 26	72 ± 26	0.58
Smoking		81 (28%)	82 (30%)	18 (25%)	0.63
Family history of HCM		13 (4%)	9 (4%)	6 (8%)	0.19
HCM signs and symptoms					
	Angina	107 (36%)	83 (30%)	29 (40%)	0.18
	Dyspnea ^e^	142 (48%)	110 (40%)	25 (34%)	0.04
	Palpitations	51 (17%)	60 (22%)	11 (15%)	0.25
	Syncope	45 (15%)	43 (16%)	8 (11%)	0.59
	Non-sustained ventricular tachycardia	17 (6%)	23 (8%)	5 (7%)	0.48
	Aborted sudden cardiac death	2 (1%)	2 (1%)	1 (1%)	0.83
Hypertension		244 (83%)	223 (81%)	63 (86%)	0.6
Diabetes mellitus ^c,f^		90 (31%)	50 (18%)	28 (38%)	<0.001
Coronary artery disease		72 (24%)	61 (22%)	12 (16%)	0.33
History of coronary artery revascularization		53 (18%)	41 (15%)	9 (12%)	0.4
Congestive heart failure		48 (16%)	35 (13%)	9 (12%)	0.42
NYHA functional class ^a,c,f^		1.8 ± 0.8	1.6 ± 0.7	1.3 ± 0.5	<0.001
NYHA functional class III/IV ^d^		56 (19%)	34 (12%)	3 (4%)	0.002
Cerebrovascular accident		33 (11%)	33 (12%)	10 (14%)	0.84
Atrial fibrillation		86 (29%)	84 (31%)	15 (21%)	0.23
Peripheral vascular disease		29 (10%)	19 (7%)	10 (14%)	0.16
Chronic obstructive pulmonary disease ^f^		26 (9%)	30 (11%)	1 (1%)	0.01
Implantable cardioverter defibrillator		26 (9%)	21 (8%)	7 (10%)	0.82
History of septal myectomy		3 (1%)	3 (1%)	0	0.68
History of alcohol septal ablation		4 (1%)	4 (1%)	0	0.59
Medications					
	Aspirin ^c^	142 (48%)	98 (36%)	33 (45%)	0.009
	ACEi/angiotensin receptor blocker	125 (43%)	106 (39%)	39 (53%)	0.08
	Beta-blocker	203 (69%)	169 (62%)	42 (58%)	0.08
	Calcium-channel blocker ^e^	81 (28%)	81 (30%)	33 (45%)	0.01
	Direct oral anticoagulant	55 (19%)	55 (20%)	12 (16%)	0.77
	Diuretics	82 (28%)	64 (23%)	16 (22%)	0.36
	P2Y12-inhibitor	56 (19%)	41 (15%)	7 (10%)	0.11
	Spironolactone	9 (3%)	10 (4%)	4 (5%)	0.61
	Statin	182 (62%)	152 (55%)	39 (53%)	0.2
	Warfarin	26 (9%)	16 (6%)	1 (1%)	0.06

ACEi, angiotensin converting enzyme inhibitor; HCM, hypertrophic cardiomyopathy; NYHA, New York Heart Association; P2Y12, purinergic receptor P2Y, G-protein coupled, 12 protein. ^a^*p* < 0.001 Hispanic versus African-American, ^b^*p* < 0.001 Non-Hispanic versus African-American, ^c^*p* < 0.05 Hispanic versus Non-Hispanic, ^d^*p* < 0.05 Hispanic versus African-American, ^e^not significant after Dunn-Bonferroni test; ^f^*p* < 0.05 Non-Hispanic versus African-American.

### 3.2 Echocardiographic Analyses

The mean LV ejection fraction of the cohort was 68 ± 11% and did not differ between the HL, NH, and AA groups. Notably, HL patients had a slightly larger LV internal diastolic diameter index (23 ± 4 vs 22 ± 4 vs 22 ± 4, *p* = 0.002), while AA patients had a greater LV mass index (255 ± 91 vs 254 ± 103 vs 287 ± 118 g/m^2^, *p* = 0.04), apical wall thickness (11 ± 4 vs 11 ± 4 vs 13 ± 4 mm, *p* = 0.01), and relative wall thickness (0.59 ± 0.15 vs 0.58 ± 0.16 vs 0.64 ± 0.26, *p* = 0.007). Interventricular septal wall thickness was slightly greater in AA versus NH patients (20 ± 6 vs 18 ± 4 mm, *p* = 0.02). The most prevalent HCM phenotype was a sigmoid septum in the HL and NH groups, while apical HCM was most prevalent amongst AA patients. Obstructive HCM was more least common in AA patients (55 vs 51 vs 37%, *p* = 0.02), with similar mean peak systolic LV outflow tract pressure gradients between ethnicities.

The peak trans-mitral E-wave velocity was lowest in the AA group (0.89 ± 0.31 vs 0.87 ± 0.26 vs 0.77 ± 0.22 m/s, *p* = 0.004), while the average E/e’ ratio was highest amongst HL patients (16.4 ± 8.1 vs 14.9 ± 6.6 vs 13.3 ± 4.5, *p* = 0.002), as measures of LV compliance and filling pressure. A signal towards a larger left atrial volume index was also observed in the HL group. Finally, moderate to severe mitral regurgitation (35 vs 23 vs 12%, *p* < 0.001) was more prevalent in the HL group. Comprehensive details of 2-dimensional echocardiographic measures are shown in Table 2.

**Table 2.** **Detailed 2-dimensional echocardiography in patients with hypertrophic cardiomyopathy according to ethnicity**.

Variable		Hispanic	Non-Hispanic	African-American	*p*-value
		N = 294	N = 274	N = 73	
Left ventricular structure and function					
	LV ejection fraction (%)	68 ± 10	67 ± 10	67 ± 12	0.43
	LV internal diastolic diameter index (mm/m^2^) ^a,b^	23 ± 4	22 ± 4	22 ± 4	0.002
	LV internal systolic diameter index (mm/m^2^) ^b^	14 ± 3	14 ± 3	13 ± 3	0.03
	LV mass index (g/m^2^) ^b,c^	255 ± 91	254 ± 103	287 ± 118	0.04
	Septal wall thickness (mm) ^c^	19 ± 4	18 ± 4	20 ± 6	0.03
	Posterior wall thickness (mm) ^b,d^	12 ± 2	12 ± 2	13 ± 3	0.001
	Apical wall thickness (mm) ^b,c^	11 ± 4	11 ± 4	13 ± 4	0.01
	Septal-to-posterior wall thickness ratio	1.6 ± 0.4	1.6 ± 0.4	1.6 ± 0.4	0.68
	Relative wall thickness ^b,c^	0.59 ± 0.15	0.58 ± 0.16	0.64 ± 0.26	0.007
	Left ventricular apical aneurysm	9 (3%)	3 (1%)	4 (5%)	0.07
Left ventricular morphology					
	Sigmoid septum ^d,e^	151 (51%)	138 (50%)	14 (19%)	<0.001
	Reverse curve	53 (18%)	45(16%)	16 (22%)	0.55
	Neutral	40 (14%)	47 (17%)	18 (25%)	0.28
	Apical ^d,e^	50 (17%)	44 (16%)	25 (34%)	<0.001
Left ventricular outflow tract					
	Obstructive hypertrophic cardiomyopathy ^b,c^	162 (55%)	141 (51%)	27 (37%)	0.02
	Peak systolic pressure gradient	66 ± 31	64 ± 30	57 ± 26	0.33
Left ventricular diastology					
	Peak transmitral E-wave velocity (m/s) ^b,c^	0.89 ± 0.31	0.87 ± 0.26	0.77 ± 0.22	0.004
	Average mitral annular velocity (m/s) ^a^	0.059 ± 0.016	0.063 ± 0.019	0.061 ± 0.016	0.01
	Average E/e’ ratio ^a,b^	16.4 ± 8.1	14.9 ± 6.6	13.3 ± 4.5	0.002
Right ventricular structure and function					
	Right ventricular basal diameter (mm)	33 ± 5	34 ± 5	34 ± 4	0.12
	Tricuspid annular plane systole excursion (mm)	18 ± 4	18 ± 4	19 ± 4	0.13
	Right ventricular systolic pressure (mmHg)	36 ± 13	36 ± 12	36 ± 15	0.99
Left atrial volume index (mL/m^2^)		40 ± 17	38 ± 15	36 ± 12	0.08
Mitral valve characteristics					
	Systolic anterior motion ^f^	178 (61%)	161 (59%)	33 (45%)	0.05
	Moderate to severe mitral regurgitation ^a,e^	103 (35%)	63 (23%)	9 (12%)	<0.001

LV, left ventricle. Right ventricular systolic pressure was available in 220 Hispanic, 189 Non-Hispanic, and 46 Black patients. ^a^*p* < 0.05 Hispanic versus Non-Hispanic, ^b^*p* < 0.05 Hispanic versus African-American, ^c^*p* < 0.05 Non-Hispanic versus African-American, ^d^*p* < 0.001 Non-Hispanic versus African-American, ^e^*p* < 0.001 Hispanic versus African-American, ^f^not significant after Dunn-Bonferroni test.

### 3.3 Clinical and Echocardiographic Correlation with NYHA Functional Class

The multivariate linear regression analysis correlating clinical and echocardiographic parameters with NYHA functional class is shown in Table 3. HL ethnicity (β = 0.1, *p* = 0.03) was found to be associated with a higher NYHA functional class independent of moderate to severe mitral regurgitation (β = 0.2, *p* < 0.001), chronic obstructive pulmonary disease (β = 0.17, *p* < 0.001), female gender (β = 0.13, *p* < 0.001), coronary artery disease (β = 0.12, *p* < 0.001), atrial fibrillation (β = 0.11, *p* = 0.004), peak trans-mitral E-wave velocity (β = 0.11, *p* = 0.004), reverse curve septal morphology (β = 0.1, *p* = 0.008), and LV mass index (β = 0.1, *p* = 0.01) (Full model, F (10, 630) = 20.7, r = 0.5, r^2^ = 0.24, *p* < 0.001). Age, obstructive hypertrophic cardiomyopathy, and left atrial volume index were excluded from the full model due to statistical non-significance.

**Table 3.** **Multi-variate linear regression analysis correlating New York Heart Association functional class with clinical and echocardiographic parameters in hypertrophic cardiomyopathy**.

Variable		Unstandardized β	95% confidence interval for β		Standardized β	*p*-value
			Lower	Upper		
Step 1						
	Constant	1.5	1.4	1.6		<0.001
	Hispanic ethnicity	0.2	0.1	0.4	0.15	<0.001
Step 2						
	Constant	0.6	0.3	0.9		<0.001
	Hispanic ethnicity	0.1	0.01	0.2	0.1	0.03
	Moderate or greater mitral regurgitation	0.3	0.2	0.5	0.2	<0.001
	Chronic obstructive pulmonary disease	0.5	0.3	0.7	0.17	<0.001
	Female	0.2	0.1	0.3	0.13	<0.001
	Coronary artery disease	0.2	0.1	0.4	0.12	<0.001
	Atrial fibrillation	0.2	0.1	0.3	0.11	0.004
	Peak transmitral E-wave velocity (m/s)	0.3	0.1	0.5	0.11	0.004
	Reverse curve septal morphology	0.2	0.05	0.4	0.1	0.008
	Left ventricular mass index (g/m^2^)	0.002	0.001	0.003	0.1	0.01
∙ Full model with significant variables. F (10, 630) = 20.7, r = 0.5, r^2^ = 0.24 (*p* < 0.001).						
∙ Age, obstructive hypertrophic cardiomyopathy, and left atrial volume index were excluded from the full model due to statistical non-significance.						
∙ All other demographic, clinical, and echocardiographic variables were statistically non-significant in univariate linear regression modeling and excluded.						

### 3.4 Clinical Outcomes

All-cause mortality occurred in 54 (8%) patients and 190 (30%) experienced a cardiovascular hospitalization, with no difference between the HL, NH, and AA groups. While the event rate was low given the sample size of AA patients, it is acknowledged that sudden cardiac death occurred more frequently in AA patients (1 vs 1 vs 7%, *p* = 0.008), with a signal towards an increased prevalence of cardiovascular mortality as well (4 vs 3 vs 8%, *p* = 0.07). Of note, septal myectomy was performed more frequently in the HL group (23 vs 12 vs 5%, *p* < 0.001). There was no difference between groups with regards to myocardial infarction, cerebrovascular accident, or hospitalization for HF, angina, or arrhythmia (Table 4).

**Table 4.** **Clinical outcomes of patients with hypertrophic cardiomyopathy according to ethnicity**.

Variable	Hispanic	Non-Hispanic	African-American	*p*-value
	N = 294	N = 274	N = 73	
Composite outcomes	105 (36%)	88 (32%)	17 (23%)	0.65
(All-cause mortality or any cardiovascular hospitalization)				
All-cause mortality	27 (9%)	19 (7%)	8 (11%)	0.45
Cardiovascular mortality	11 (4%)	7 (3%)	6 (8%)	0.07
Sudden death ^a,b^	4 (1%)	4 (1%)	5 (7%)	0.008
Myocardial infarction	13 (4%)	12 (4%)	6 (8%)	0.36
Cerebrovascular accident	20 (7%)	25 (9%)	6 (8%)	0.59
Any cardiovascular hospitalization	94 (32%)	76 (28%)	20 (27%)	0.49
Heart failure hospitalization	48 (16%)	31 (11%)	9 (12%)	0.21
Angina hospitalization	44 (15%)	41 (15%)	11 (15%)	1
Arrhythmia hospitalization	26 (9%)	24 (9%)	6 (8%)	0.99
Septal myectomy ^a,c^	69 (23%)	32 (12%)	4 (5%)	<0.001
Alcohol septal ablation	4 (1%)	7 (3%)	1 (1%)	0.55

^a^*p* < 0.05 Hispanic versus African-American, ^b^*p* < 0.05 Non-Hispanic versus African-American, ^c^*p* < 0.05 Hispanic versus Non-Hispanic.

## 4. Discussion 

**Fig. 1.**
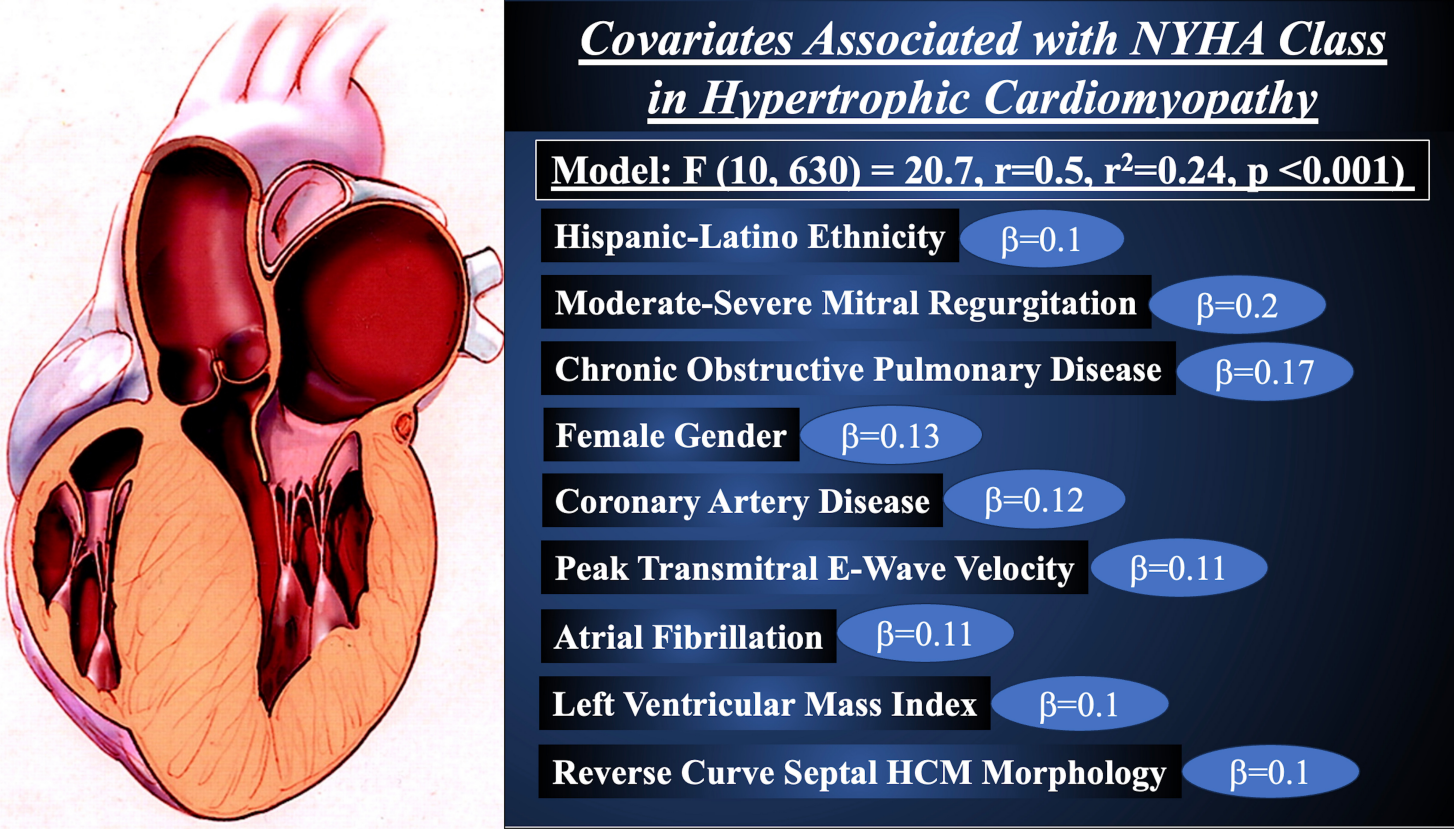
**Covariates Associated with New York Heart Association Functional Class in Hypertrophic Cardiomyopathy**.

In this retrospective study of 641 HCM patients stratified by HL, NH, and AA race, the following salient findings were noted: (1) HL patients had a greater prevalence of female gender when compared with NH, and more NYHA class III/IV symptoms than AA; (2) a higher NYHA functional class was reported by the HL group, with higher LV filling pressures; (3) a sigmoid septum HCM phenotype was most common in the HL and NH population, while AA had more apical HCM a greater LV mass and wall thickness, and less obstructive HCM physiology; (4) moderate to severe mitral regurgitation was most prevalent amongst HL patients; (5) no difference was observed between groups at 2.5-year follow-up in regards to all-cause mortality or cardiovascular hospitalization; and, (6) HL ethnicity was associated with a higher NYHA functional class independent of moderate to severe mitral regurgitation, chronic obstructive pulmonary disease, atrial fibrillation, peak trans-mitral E-wave velocity, female gender, coronary artery disease, LV mass index, and reverse curve septal morphology (Fig. 1).

Heart failure with preserved or reduced LV ejection fraction remains a global leading cause of morbidity and mortality [13]. According to the Heart Failure Society of America, the prevalence of HF in American adults is expected to increase to 9 million by the year 2030, which is being mirrored by worsening rates of HF-related hospitalizations and mortality [13]. The estimated cost burden of HF on the United States economy is 70 to 160 billion dollars per year, with recognized disparities in clinical presentation and resources between racially and ethnically diverse populations [14]. In patients with HCM, progressive HF symptoms are observed in approximately 30% over mid-term follow-up, the onset of which has been shown to increase the risk of advancement to end-stage cardiomyopathy, major adverse cardiovascular events, and significantly impacts quality of life metrics [15, 16]. Regardless of preserved or reduced LV ejection fraction, the yearly cost per HCM patient approaches $35,000 dollars and affects both tertiary referral and ‘real-world’ outpatient clinical settings [17]. Our findings of HL race in HCM being associated with worse HF symptoms and functional status is salient in that it identifies a subgroup of patients that may possibly benefit from earlier or more aggressive risk stratification, medical therapy, or structural intervention.

In comparison with AA individuals, HL and NH patients were slightly older at diagnosis in the present study, and when compared with previous literature [18]. Furthermore, the majority of HL patients were female, who accounted for over half of the cohort participants. Elderly patients with HCM have a complex clinical profile with significant traditional cardiovascular co-morbidities, and a 50% prevalence of all-cause mortality or appropriate internal cardioverter defibrillator discharge at mid-term follow-up [19]. Women with HCM have historically been diagnosed later in the disease course and with more advanced HF symptoms [20]. Importantly, the association of HL ethnicity with higher NYHA functional class was independent of demographic, clinical, and anatomic echocardiographic variables, including age and female gender. Finally, an important mediator of health outcomes in HCM, which was not captured in our institutional registry as a distinct characteristic variable, was socioeconomic status. Disadvantaged and minority HCM patients experience worse health-quality metrics, have more advanced symptomatology, and lower medical therapy adherence [21]. Thus, whether our observations regarding the co-existence of higher-risk characteristics within the HL cohort is a consequence of genetic variability in expression or under-diagnosis due to health care disparities unaccounted for needs to be further examined. Reflecting these points is the regression model’s relatively low explanatory power (r^2^ = 0.24), suggesting that indeed unmeasured confounders may contribute to the observed disparities. Future in-depth studies on the impact of co-morbid conditions as they relate to the clinical course of HCM across racial groups are needed to explore potential confounding.

Septal morphology is an important determinate of clinical presentation and adverse outcomes in patients with HCM, with the sigmoid septum and reverse curve morphologies accounting for nearly 70% of patients, and neutral septum or apical HCM found in the remaining cases [9]. In our study, the prevailing phenotype amongst HL patients was a sigmoid septum. Sigmoid phenotype is associated with more LV outflow tract obstruction, a greater degree of mitral valve SAM, and more severe mitral regurgitation [22, 23]. Indeed, 61% of the HL patients studied herein had significant mitral valve SAM and 35% experienced moderate to severe mitral regurgitation, with the latter being significantly more prevalent in HL versus NH or AA individuals. Consequently, septal myectomy was performed more frequently in the HL group, which is antithesis to prior published data reporting far fewer septal reduction procedures performed in HL and AA patients when compared with NH Whites [24]. The authors of these prior investigations suggested a role of implicit and provider bias as important confounders. In our medical center, a diverse physician workforce that cares for a predominantly HL population helps to ameliorate some of these disparities. In addition, septal reduction procedures are performed in patients with limiting symptoms as evidenced by worse NYHA functional class, as was observed in our HL cohort [25].

There are study limitations and caveats that should be considered when interpreting the present data. First, the study design was retrospective which carries and inherent patient selection bias. Additionally, AA patients comprised only 11% (N = 73) of the cohort, which may induce an overestimation of effect size or invariably a type II statistical error. Second, the study groups were stratified as HL, NH, and AA based on patients’ self-identification, thus introducing possible subjectivity in the cohort. Additionally, the NH group was comprised of patients of multiple ethnicities other than HL and AA, which results in intra-group heterogeneity. Whether these aspects of the study design impacted the HCM prevalence we adjudicated is not known. Third, there were limited resources available for cardiac magnetic resonance imaging, which generally precludes definitive assessment for myocardial fibrosis and scar. This is of particular importance given the role of late gadolinium enhancement and myocardial fibrosis in the diagnosis and risk stratification of HCM patients. Similarly, there was no information available on genetic testing or previously confirmed pathogenic gene mutations. Given that a family history of HCM lowers the diagnostic threshold for HCM, this may have underestimated the true prevalence in the present study. This most often was a result of the tertiary referral pattern of our program, and socioeconomic or geographical challenges for patients, the latter which as discussed was not accounted for in the study design and institutional registry. Fourth, as previously mentioned there was a higher incidence of sudden cardiac death in the AA group when compared with HL and NH patients. The overall event rate for this outcome was 2% (N = 13) across the three groups, and thus, is best interpreted as hypothesis-generating given the substantial risk of type I statistical error. Fifth, our study was a single tertiary care center analysis which may introduce uncontrollable confounding due to selection biases associated with specific geographical settings. Nevertheless, the location of our institution allows for a population of both advantaged and underserved populations, particularly from Central and South America and the Caribbean, which provides a unique study cohort. Sixth, the older age and cardiovascular risk profile of patients in HL and NH likely reflects the later diagnosis in patients immigrated from Central and South America, and the Caribbean, many of whom did not have access to adequate medical care for diagnosis and therapy of their HCM until the 5th to 7th decades of life. Additionally, prior landmark epidemiologic registry studies on HCM have often been collated from large centers with dedicated and specialized Heart Failure, Cardiomyopathy, and/or HCM programs. These programs receive early referrals, tend to be located in larger socioeconomically developed and populated communities, and are supported by comprehensive clinical resources, which impacts the cohort demographic and clinical characteristics. It is prudent to note that epidemiological data have also shown the robust increase in age at diagnosis, as was presented in the multinational Sarcomeric Human Cardiomyopathy Registry of 7286 HCM patients [25]. It is hypothesized that the widespread adoption of electrocardiographic and echocardiographic screening in communities has fostered the physician awareness of asymptomatic HCM patients and improved the diagnostic yield in lieu of genetic testing. This may also explain our findings of a younger age in AA patients, in whom apical HCM phenotype was most common and known to have marked electrocardiographic abnormalities. Seventh, nearly 20% of our cohort had apical HCM, where the thickest LV segments would be located at the distal lateral LV and at the apex. The average LV wall thickness and relative wall thickness included all morphologies/phenotypes averaged together, and thus, we believe this may have attenuated the final measurements presented. Similarly, care should be taken in interpreting the reported LV mass in our cohort, which is often spurious in asymmetric LVs seen in HCM patients. Finally, although the patient mid-term follow-up in our study was 100% complete at a median time of 2.5-years, the outcomes observed should be placed within this time context. As the natural history of HCM and treatment options have significantly improved over contemporary practice, continued surveillance with accruing follow-up is needed to appropriately and confidently interpret the clinical outcomes, and external validation of our findings is of paramount importance.

## 5. Conclusions

In conclusion, HL ethnicity in HCM is associated with worse heart failure symptoms and functional class, and more mitral regurgitation, when compared with NH and AA patients. These findings characterize a subgroup of patients that may possibly benefit from earlier or more aggressive risk stratification and treatment. The association of HL ethnicity with higher NYHA functional class was independent of established demographic and clinical variables.

## Availability of Data and Materials

Due to institutional review board and ethical regulations, all publicly available data are contained within the article.
